# Supplementary material for: An optogenetic toolbox for unbiased discovery of functionally connected cells in neural circuits
Source: Nat Commun. 2017 Jul 24;8:116. doi: 10.1038/s41467-017-00160-z (PMC5524645; doi:10.1038/s41467-017-00160-z)
Supplement: Supplementary file 1 — Supplementary Information [file 41467_2017_160_MOESM1_ESM.pdf]

File Name: Supplementary Information
Descriptions: Supplementary Figures and Supplementary References

File Name: Peer Review File
Descriptions:

File Name: Supplementary Movie 1
Descriptions: Stimulation of ChrimsonR-expressing cells evokes behavior in zebrafish. A 5 dpf larva expressing ChrimsonR-tagRFP in nMLF cells is head-embedded in agarose, with its tail free to move. A 50 µm optic fiber targets 638 nm light (0.1 mW) to the region of the nMLF and is moved in a vertical direction. Light exposure starts at 2.6 s and evokes a tail steering response. See Supplementary Figure 2 for details.

File Name: Supplementary Movie 2
Descriptions: 3D reconstruction of Optobow-n expression in tectal cells. Filament tracings show a descending axon of the ChrimsonR-expressing cell (magenta), a bistratified morphology of the first connected cell (yellow), and a contralaterally projecting axon of the second connected cell (orange). Anterior is left, lateral is up. See Figure 2 for details.

File Name: Supplementary Movie 3
Descriptions: 3D reconstruction of a reference brain including functionally connected cell pairs from seven individual fish. Volume rendering of the reference brain shows DAPI staining (blue), and orthogonal slicer shows DAPI (blue) and HuC (red) staining. See Figure 8 for details.

Supplementary Figure 1

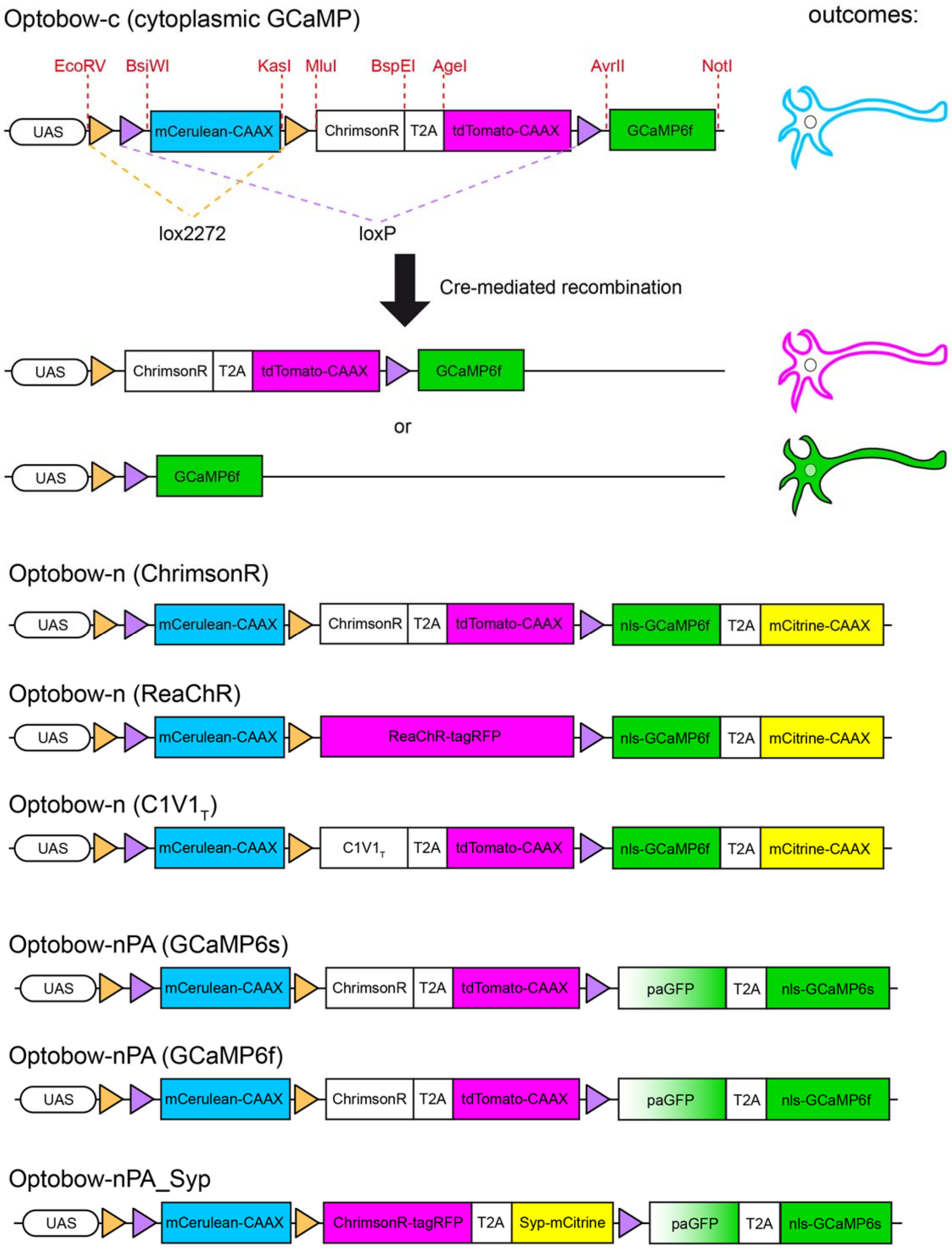

Supplementary Figure 1. Optobow enables stochastic and mutually exclusive expression of optogenetic actuator and indicator. Schematic of available Optobow constructs. Expression is dependent on the Gal4-UAS system. The open reading frames for mCerulean,

ChrimsonR-T2A-tdTomato and GCaMP6f are separated by loxP or lox2272 sites and are each followed by polyadenylation signals. mCerulean and tdTomato are both membrane-targeted (CAAX motif). Sketches for the different expression outcomes are depicted on the right. Unique restriction sites for easy replacement of open reading frames are shown in red. The Optobow-n constructs featuring the optogenetic actuators $C1V1_T$ or ReaChR (although not described in the text) have been tested successfully for functional connectivity mapping.

Supplementary Figure 2

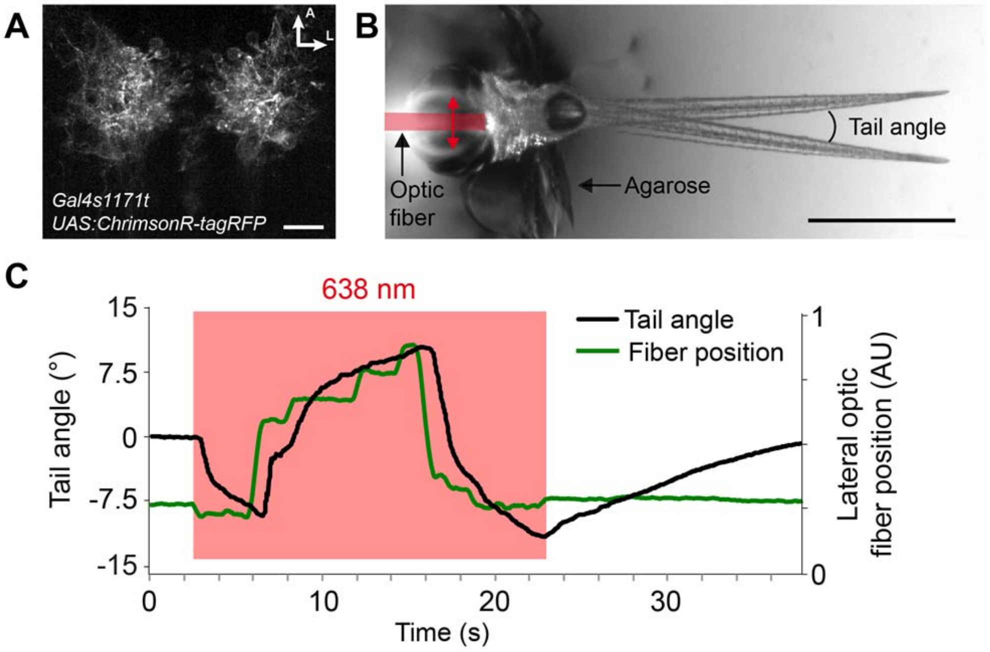

Supplementary Figure 2. Stimulation of ChrimsonR-expressing cells elicits behavior in larval zebrafish. (A) Transgenic ChrimsonR-tagRFP expression in the nucleus of the medial longitudinal fasciculus (nMLF) of a 5 dpf zebrafish larva. Scale bar, 20 µm. **(B)** Optogenetic setup as published previously[1]. The head of the same fish from (A) is embedded in agarose with its tail freed. A 50 µm optic fiber targets 638 nm light (0.1 mW) onto the region of the nMLF and is moved in a vertical direction. A projection of the initial and maximum left-deflected tail positions is shown. Scale bar, 1 mm. **(C)** Measurement of the tail angle and the fiber position over time of the fish shown in (B). The red rectangle depicts the epoch of 638 nm light stimulation.

Supplementary Figure 3

Construct	Fish line	Expression (w/o Cre)	Variegation
Optobow-c	*Tg(UAS:Optobow-c)mpn135*	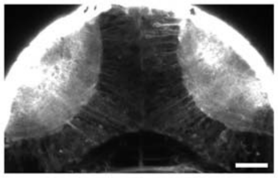	weak
Optobow-c	*Tg(UAS:Optobow-c)mpn136*	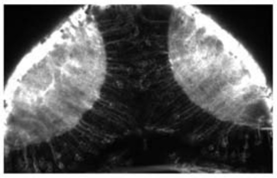	medium
Optobow-n	*Tg(UAS:Optobow-n)mpn137*	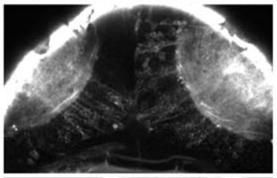	medium
Optobow-n	*Tg(UAS:Optobow-n)mpn138*	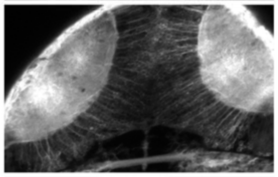	weak
Optobow-nPA (nls-G6s)	*Tg(UAS:Optobow-nPA)mpn141*	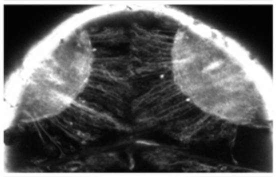	medium
Optobow-nPA (nls-G6s)	*Tg(UAS:Optobow-nPA)mpn139*	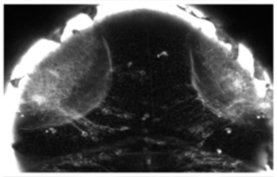	strong
Optobow-nPA (nls-G6f)	*Tg(UAS:Optobow-nPA)mpn140*	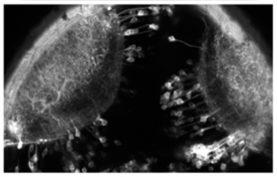	medium

Supplementary Figure 3. List of available Optobow zebrafish lines. Variegation of the different UAS-transgenic lines was scored based on expression of mCerulean-CAAX from the pan-neuronal driver *Gal4s1101t.* Scale bar: 40 µm.

Supplementary Figure 4

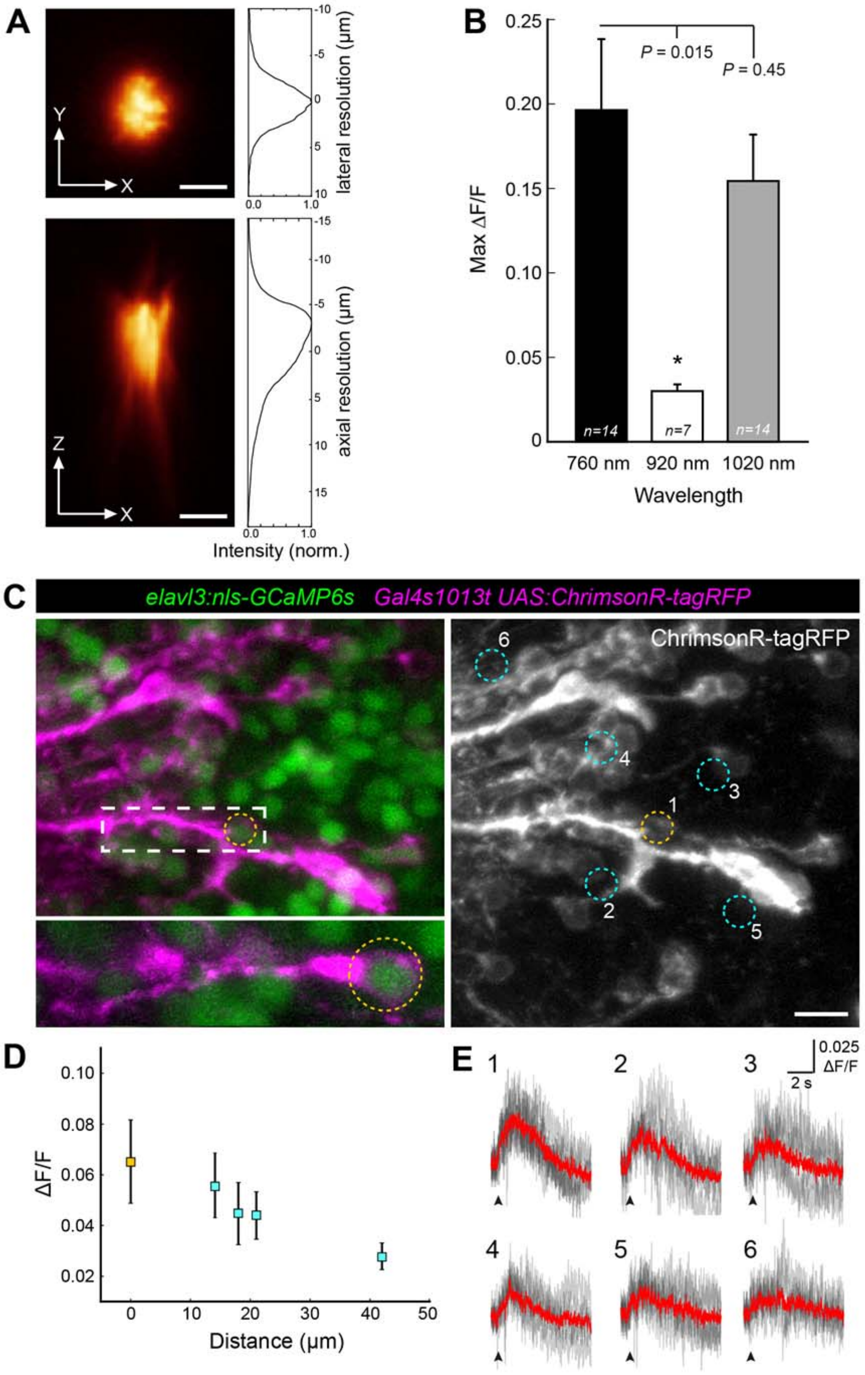

Supplementary Figure 4: Characterization of 2P holography photostimulation. (A) Axial (top) and lateral (bottom) extension of a circular illumination pattern with a diameter of 6 µm. 960 nm light was used to excite a thin layer of Fluorescein solution (0.2 mW µm^{-2}). Axial and

lateral intensity profiles are shown on the right. Scale bar, 5 µm. **(B)** Maximum calcium responses obtained upon photostimulation of ChrimsonR-expressing cells using different wavelengths. Excitation of Chrimson at 760 nm resulted in relatively large responses, likely driven by a single photon absorption mechanism. Data obtained from Optobow-nPA experiments in tectal cells. Error bars indicate SEM. **(C-E)** Characterization of ChrimsonR photostimulation selectivity *in vivo.* **(C)** Maximum projection of a confocal stack showing tectal cells in a 6 dpf zebrafish larva with a dense expression of ChrimsonR-tagRFP (left: magenta, right: white) and pan-neuronal nls-GCaMP6s (green). A close-up single confocal slice of the targeted cell is shown below the projection. A 6 µm-diameter excitation spot was used for on-target (orange circle, #1) or off-target (cyan circles, #2-6) photostimulations at 1020 nm (200 ms), while calcium responses of cell#1 were recorded simultaneously. Scale bar, 10 µm. **(D)** Averaged maximum $\Delta F/F$ calcium responses obtained from on-target and off-target stimulations as a function of distance between the recorded cell (#1) and the excitation spot. Error bars are SEM. **(E)** Raw (gray) and averaged (red) $\Delta F/F$ profiles of six excitation trials for the different spot positions indicated in (C). Stimulation time points are indicated by arrowheads.

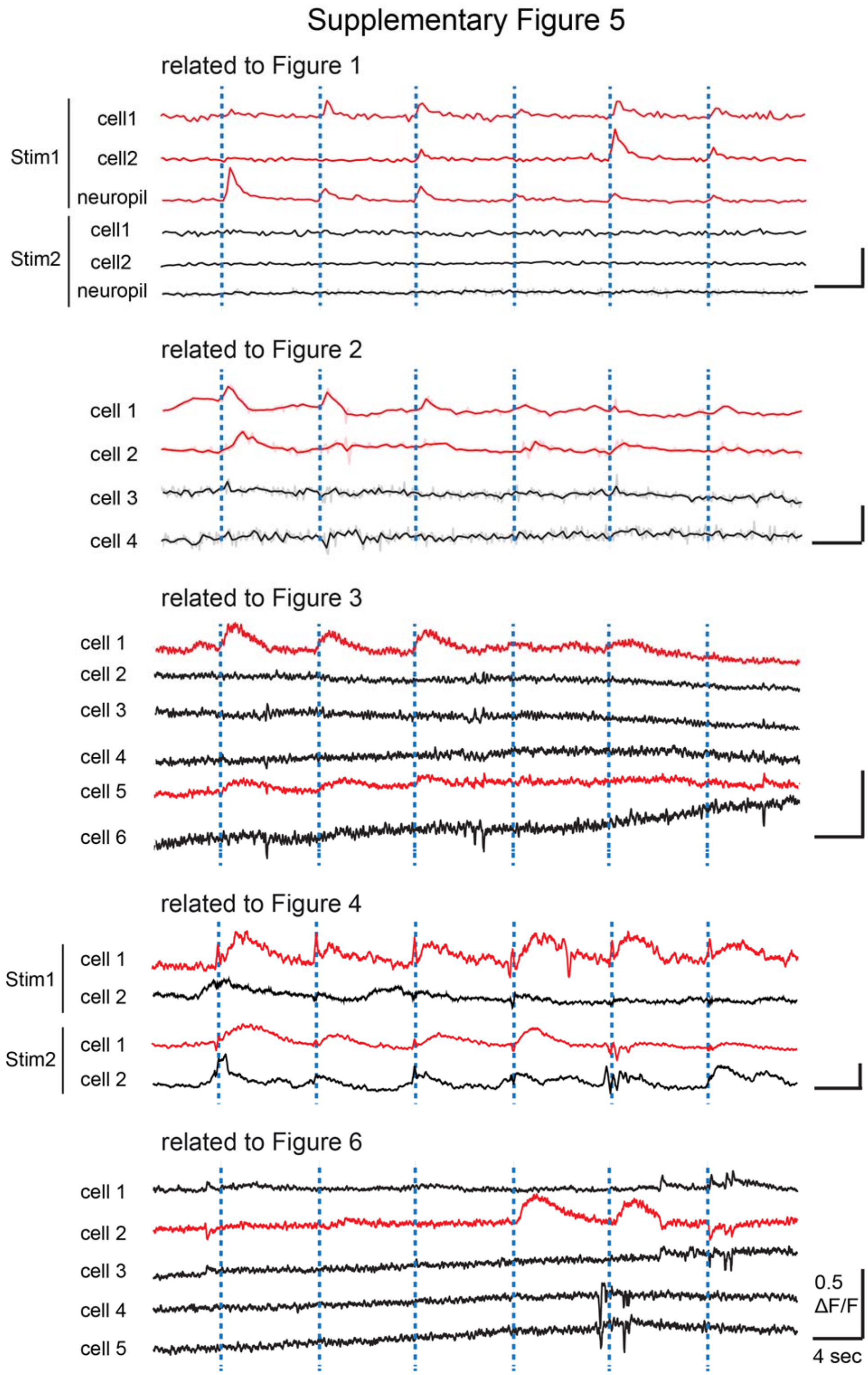

Supplementary Figure 5. Calcium measurements displayed as ΔF/F profiles. Photostimulation events are indicated by dashed blue lines. Raw, as well as averaged (dark colours) transients are shown. In Figure 3, stimulation events no. 1, 2, and 3, and in Figure 6, stimulation events no. 3, 4, and 5 are shown.

Supplementary Figure 6

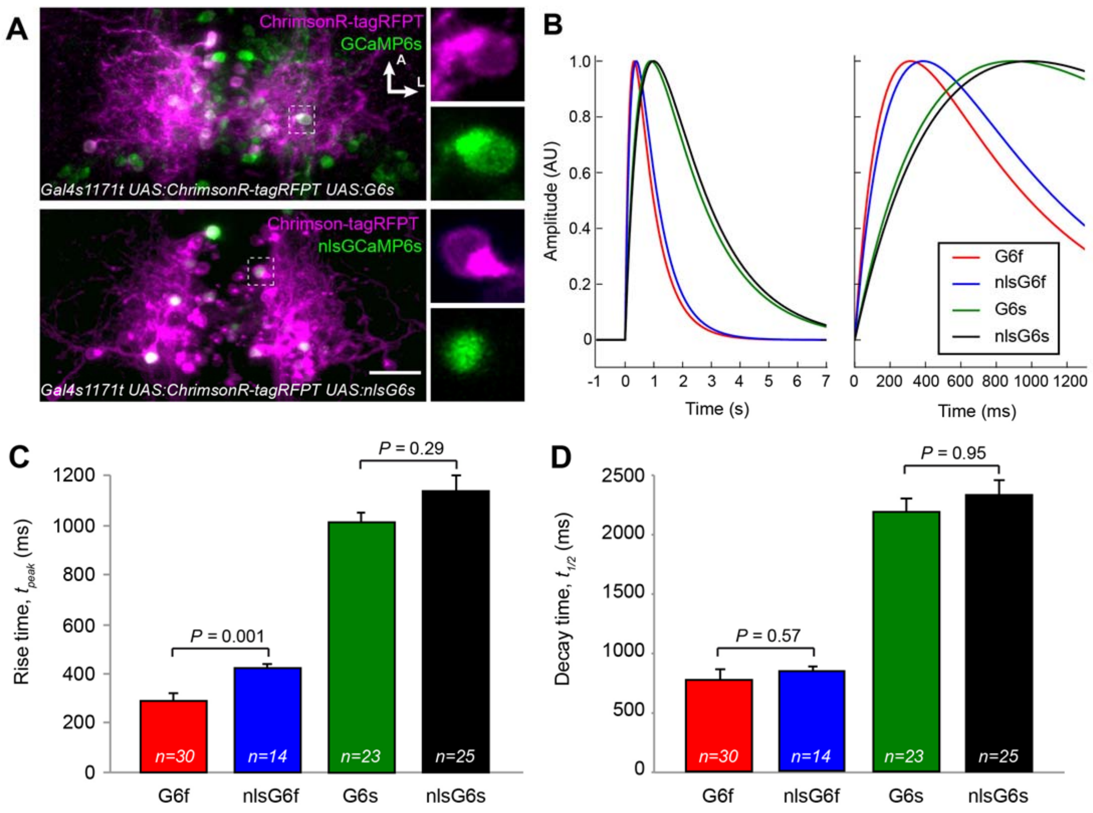

Supplementary Figure 6. Comparison of cytoplasmic and nuclear GCaMP6 variants. **(A)** GCaMP6 dynamics were measured in nMLF cells *(Gal4s1171t)* transiently expressing ChrimsonR-tagRFP and either of the GCaMP6 variants. Single cells were photostimulated with 200 ms of 760 nm light and the GCaMP transients were measured by ~300 Hz line scans across the soma of the stimulated cell. Single-channel closeups shown on the right of the regions indicated by dashed boxes, show membrane, cytoplasmic, or nuclear localization of ChrimsonR-tagRFP, GCaMP6s or nls-GCaMP6s, respectively. G6s, GCaMP6s; G6f, GCaMP6f. Scale bar, 30 μm. **(B)** Normalized mathematical fit for the fluorescence responses of all measured GCaMP6 versions. Number of trials (n) is indicated in (C) and (D). **(C)** Comparison of fluorescence rise times to peak intensity. **(D)** Comparison of half decay times. Error bars indicate SEM.

Supplementary Figure 7

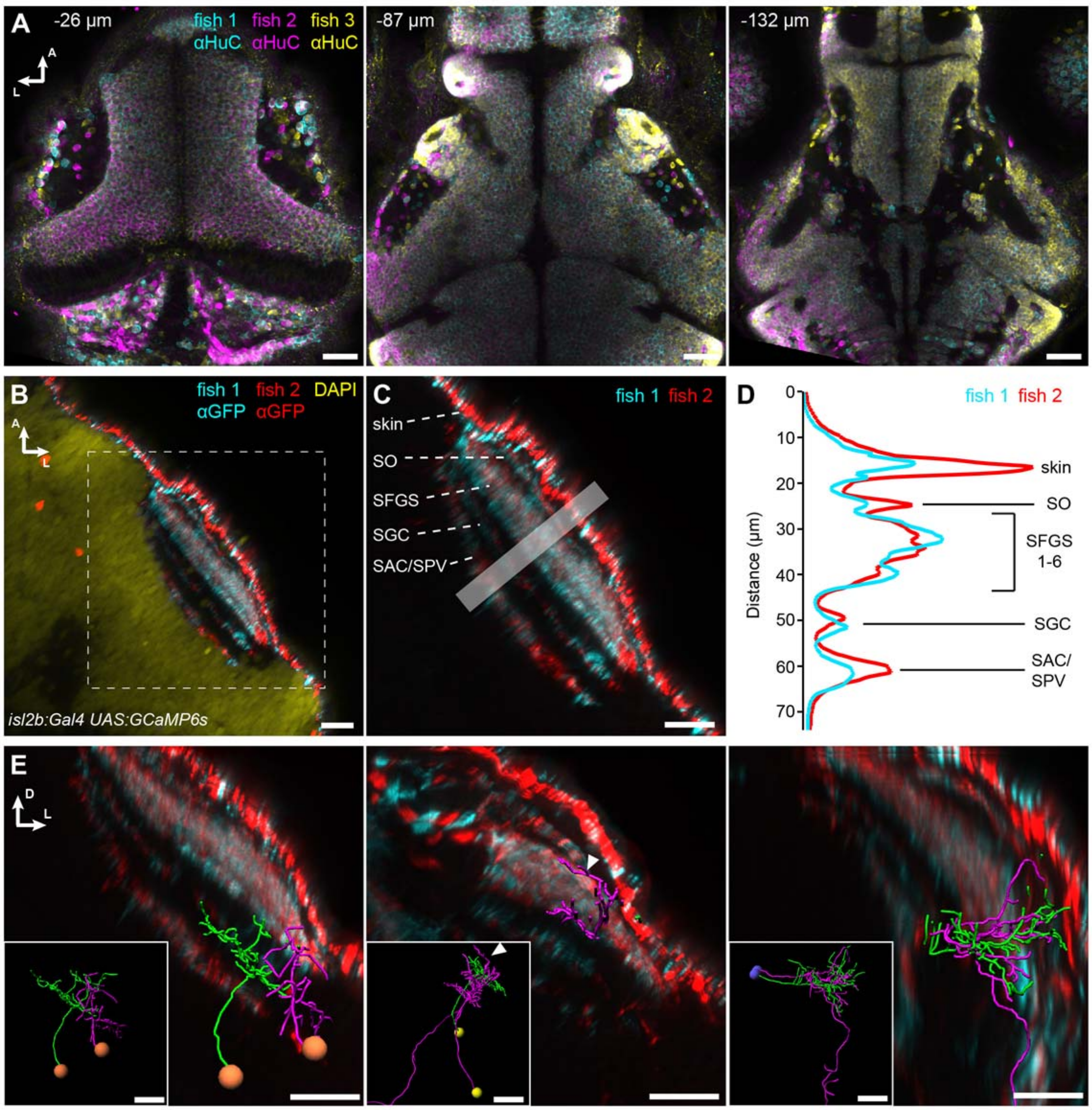

Supplementary Figure 7. Accuracy of registration and addition of anatomical reference patterns. (A) Three 5 dpf Optobow-nPA-expressing larvae stained for HuC have been registered into one reference brain. Note the accuracy of registration at different z levels (labeled as distance from dorsal skin in µm). Scale bar, 30 µm. **(B)** Two 5 dpf larvae expressing GCaMP6s under control of *isl2b:Gal4* (stained for GFP) were co-registered into the reference brain. Scale bar, 20 µm. **(C)** A closeup of the tectal neuropil shows the different innervation strata of RGC axons. SO, stratum opticum; SFGS, stratum fibrosum et griseum superficiale; SGC, stratum griseum centrale; SAC/SPV, stratum album centrale/stratum

periventriculare. Scale bar, 20 µm. **(D)** Intensity profiles through the boxed region in (C) show the alignment accuracy of RGC innervation strata. **(E)** Examples of three co-registered cell pairs and their dendritic and axonal arborizations in specific tectal layers. Insets taken from Figure 8. Scale bar, 20 µm.

Supplementary References

1. Thiele, T. R., Donovan, J. C. & Baier, H. Descending control of swim posture by a midbrain nucleus in zebrafish. *Neuron* **83,** 679–691 (2014).
